# Combined Esophageal Intraluminal Impedance, pH and Skin Conductance Monitoring to Detect Discomfort in GERD Infants

**DOI:** 10.1371/journal.pone.0043476

**Published:** 2012-08-23

**Authors:** Francesco Cresi, Emanuele Castagno, Hanne Storm, Leandra Silvestro, Roberto Miniero, Francesco Savino

**Affiliations:** 1 Department of Pediatrics, University of Turin, S.Anna - Regina Margherita Children’s Hospital, Turin, Italy; 2 Division of Pediatric Emergency, S. Anna -Regina Margherita Children’s Hospital, Turin, Italy; 3 Skill Simulation lab, Medical faculty, University of Oslo, Oslo, Norway; 4 Department of Internal Medicine and Surgery, University “Magna Graecia”, Catanzaro, Italy; University of Florida, United States of America

## Abstract

**Background:**

The clinical significance of weakly acidic reflux in infants is unclear. Skin conductance is a novel not-invasive method to evaluate discomfort. The aim of our study was to evaluate reflux-induced discomfort in infants with gastroesophageal reflux disease using simultaneously combined skin conductance and esophageal multichannel intraluminal impedance and pH monitoring.

**Methodology/Principal Findings:**

Infants with gastroesophageal reflux symptoms were investigated for almost 20 hours divided into 120-second intervals. Temporal relationships between refluxes and discomfort were evaluated calculating the symptom association probability. Twelve infants aged 17–45 days were studied. Out of 194.38 hours of adequate artifact-free MII/pH and skin conductance monitoring, 584 reflux events were observed; 35.78% were positive for stress, of which 16.27% were acid and 83.73% weakly acidic. A significant association between refluxes and discomfort (*p*<0.05) was present in all infants. The intervals with reflux events showed increased skin conductance values compared to reflux-free intervals (*p*<0.001); SC values were similar for acid and weakly acidic reflux events.

**Conclusion/Signficance:**

Discomfort was significantly associated with reflux events and did not differ between weakly acidic and acid refluxes. Our results may raise concerns about the over-prescription use of antacid drugs in the management of gastroesophageal reflux symptoms in infancy.

## Introduction

Gastroesophageal reflux (GER) is a common and transient condition of infancy, defined as the passage of gastric content into the esophagus. When GER becomes clinically relevant as a result of the quality and quantity of the refluxate, the disturbance can be defined as gastroesophageal reflux disease (GERD) [1], [2]. The pathogenesis of GERD is multifactorial and not yet fully understood [3], [4]. Transient lower esophageal sphincter relaxation plays a key role, but increased gastric acidity, lower esophageal sphincter hypotonia, impaired esophageal clearing mechanisms, and delayed gastric emptying may also be involved [5], [6].

Combined esophageal multichannel intraluminal impedance and pH (MII/pH) monitoring detects reflux events as temporary changes in impedance recorded between pairs of electrodes located along an esophageal probe, and determines their pH by means of a pH-sensitive antinomy electrode. Rosen and colleagues reported that the addition of MII to standard pH monitoring results in a change in management in approximately 25% of infants, and the role of MII/pH has been well established in clinical practice [7]. The technique permits the identification of reflux events independently of their pH, and defines in deeper detail the characteristics of GER at different ages. GER shows peculiar features particularly in infancy, such as high frequency of reflux events, and prevalence of weakly acidic reflux events (pH 4–7) that cannot be identified by pH monitoring alone. The clinical significance of these weakly acidic reflux events remains unclear [8]. Their possible role in the pathogenesis of GERD in the first months of life is controversial and, if confirmed, could induce a change in the diagnostic and therapeutic management of the disease.

Skin Conductance (SC) maybe considered, to our knowledge, a new specific physiological indicator of pain, which is only influenced by emotions and not by changes in blood volume or respiration, fever, hypothermia, medication acting on blood circulation, neuromuscular blockers [9]–[13]. Recently SC monitoring has been reported to be a valid, non-invasive, physiological measure of discomfort in both preterm and term infants [14]–[16]. SC monitoring measures emotional sweating due to skin sympathetic nerve activity after painful stimuli, which is then used as an indicator of stress and discomfort [14], [17]–[21] sweat production at the palm and sole of the foot increases upon skin sympathetic activation of the palmar and plantar eccrine sweat glands after pain and discomfort. When a skin sympathetic nerve burst occurs, sweat is released onto the surface of the skin and an increase in conductance occurs, then the sweat is reabsorbed and the conductance decreases, leading to a SC peak. The main measure of stress in SC monitoring is peaks/second, where a peak is defined as a minimum followed by a maximum in SC values. Gestational and postnatal age have not been found to influence this measure of stress [14], [16], [22]. Furthermore, very little variation has been found in peaks/second values between and within infants at the same stress/discomfort level. Because SC responses are induced by acetylcholine acting on muscarinic receptors, they are not affected by changes in blood volume [10], [23], medications that affect blood circulation such as beta blockers, neuromuscular blockers, or environmental temperature [11]. Neither do changes in respiratory rhythm influence SC responses, including apnea [12], [13].

The aim of this study was to evaluate possible GER-induced discomfort by use of simultaneously combined MII/pH and SC monitoring in a group of infants with GERD.

## Methods

### Ethics Statement

The investigation was performed in accordance with the Declaration of Helsinki [24], [25] and the research protocol was approved by the Ethical Committee of OIRM-S.Anna - Ordine Mauriziano di Torino. Written informed consent was obtained from the mother and father of all participating infants before combined MII/pH and SC monitoring was performed.

### Patients

Combined MII/pH and SC monitoring was performed at the Neonatal Care Unit of the Regina Margherita Children’s Hospital (Turin, Italy) on infants diagnosed with GERD admitted between June 2009 and December 2010.

Inclusion criteria were: age 2–12 weeks; post conceptional age 38–46 weeks; birth weight appropriate for gestational age; symptoms of GERD (excessive regurgitations, vomits, blenking, eccessive crying, irritability, nocturnal awakenings, difficulties in feeding) according to the European and North American Societies for Pediatric Gastroenterology, Hepatology and Nutrition Guidelines on GER [3], [4]. Infants could be either breast-fed, formula-fed or mixed-fed.

Exclusion criteria were: any intake of medications with gastrointestinal effects (e.g., prokinetics, antacids, H2 receptor blockers, or proton-pump inhibitors) during the 2 weeks before recruitment; any intake of medications affecting SC (e.g., high dose atropine, central sympathetic inhibitors, or acetaminophen) during the 2 weeks before recruitment; any sign or symptom of infection, metabolic or gastrointestinal disease other than GERD, or central nervous system disease.

A detailed report of infants’ symptoms was obtained from their parents upon enrollment. Data on pregnancy, delivery mode, birth weight and length, frequency and quantity of feeding, drug consumption by infants and their mothers, and family history of gastrointestinal disorders were also recorded.

### Techniques

#### MII/pH monitoring

Single-use MII/pH catheters with a pH-sensitive antimony electrode and seven integrated impedance electrodes were used; the probe was tested and calibrated for each individual infant before insertion. The catheter was placed transnasally after a 3-hour fasting period. The pH electrode of the catheter was placed 1.5 cm up the lower esophageal sphincter [26] and its correct location was checked by extemporaneous fluoroscopy. The catheter was then taped to the infant's nose and connected to an exterior impedance-voltage transducer and an exterior recording device (Sleuth System, Sandhill Scientific Inc., Highlands Ranch, Colorado, USA), for signal processing and recording, by thin wires inside the plastic probe. Impedance was measured bipolarly between adjacent impedance electrodes, thus the seven electrodes formed six impedance channels. The distance between adjacent electrodes was 1.5 cm. The total measuring segment extended from 1.5 cm above the lower esophageal sphincter (channel 6, distal) to the pharynx with the pH sensor at channel 6.

Impedance decreases during the passage of a high-conductivity bolus, such as saliva, milk or gastrointestinal secretions, and increases during the passage of air. MII monitoring shows the patterns of antegrade and retrograde bolus movements, their duration and proximal extent [26], [27]. A reflux event is defined as a decrease in impedance starting at channel 6, extending proximally over two or more sequential channels, and followed by an increase in impedance to baseline values.

MII/pH monitoring identifies reflux events and illustrates their time of occurrence, duration, proximal extent and pH. MII/pH monitoring also detects reflux events that pH monitoring alone does not, owing either to their short duration (<15 sec), or to gastric hypoacidity (pH≥4). It thus allows the recording of reflux events that occur when the intraesophageal pH is stable. For the purposes of the present study, the duration of a reflux event was defined as the time (in seconds) between its onset at the 50% drop in impedance from baseline relative to nadir, and bolus exit at the 50% recovery point from nadir to baseline recorded at the most distal channel. Its proximal extent was defined as the number of channels sequentially involved in the temporary impedance decrease. The reflux pH was the nadir esophageal pH recorded during the event. Based on these values, the reflux events were classified as acidic (pH<4), weakly acidic (pH 4–7) and weakly alkaline (pH>7).

#### SC monitoring

Stress events were identified by changes in SC values as detected by a Stress Detector™ (Med-Storm Innovation AS, Oslo, Norway). Three disposable electrodes were fixed to the left plantar surface of the foot. The measuring electrode was placed beneath the ankle on the plantar surface, the counter current electrode was placed on the medial side of the foot on the plantar surface, and the reference voltage electrode was placed on the dorsal side of the foot and ensured a constant applied voltage of 50 mV across the stratum corneum. An amplifier contained in the Stress Detector™ converted the current through the measuring electrode to a voltage, and a synchronous rectifier was used to obtain SC values from that signal. SC values were sampled with a frequency of 50 Hz and 12-bit resolution. Data were stored on-line using a portable computer and were analyzed off-line using software that was custom designed to read the ASCI-coded files generated by the Stress Detector™. An SC peak was defined as a minimum followed by a maximum in SC values; the threshold was set at 0.02 µSiemens to avoid electrical artifacts. The number of peaks/second was used as the main indicator of stress and was expressed in Hertz (Hz). The forcefulness of the peaks was measured by the relative area under the peaks as area huge peaks (AHP) and area small peaks (ASP). An AHP is defined as the cumulative difference between the SC values recorded and an established horizontal baseline, such as the minimum point of the first peak in a selected interval. An ASP is defined as the cumulative difference between the SC values recorded and a line drawn between the minimum points of two adjacent peaks in a selected interval. Both AHP and ASP were expressed in µSiemens seconds (µSs).

#### Combined MII/pH and SC monitoring and analysis

**Figure 1:**
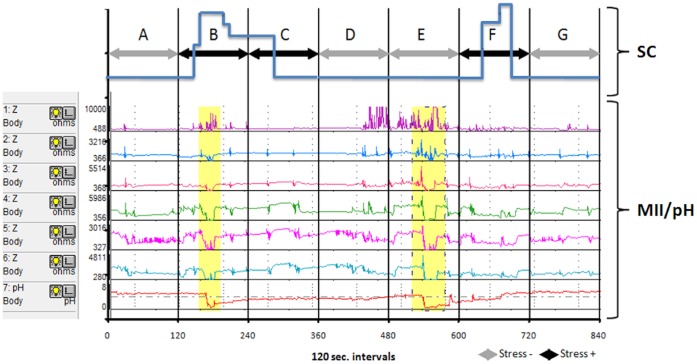
Example of combined MII/pH and SC monitoring. Capital letters A to G represent seven consecutive 120-second intervals. The upper blue line represents the SC signal; black and gray arrows indicate stress-positive and stress-negative intervals, respectively. The lower red line represents pH monitoring; each of the six colored lines in the middle indicates one of the six MII channels (starting from the top at channel 1); the yellow vertical stripes indicate reflux events. The intervals A, D and G are negative for the both presence of reflux and stress. The interval B is positive for both reflux and stress. The intervals C and F are positive for stress only. In the interval E, a stress-negative reflux occurs.

The Stress Detector™ and the MII/pH device were exactly synchronized immediately before MII/pH catheter placement. After the location of the pH-sensitive antimony electrode was checked, the SC electrodes were placed on the infant and connected to the Stress Detector™, and simultaneous recording began. The study was conducted in a quiet, half-dark room under parental or nursery care; the room temperature was constantly ranging between 21 and 23°C. Infants were kept supine and wrapped in a blanket; handling and stimulation were restricted to routine feeding and cleaning and a pacifier was occasionally used to calm crying. MII/pH data were evaluated using the BioVIEW analysis software (Sandhill Scientific Inc., Highlands Ranch, Colorado, USA). MII/pH data were visually reviewed by the same expert operator and a list of impedance-detected reflux events, including data on time, duration, proximal extent and pH, was created for each infant. Only time periods that were valid for both MII/pH and SC monitoring were combined and considered for further analysis (Figure 1). Any time period with artifacts in either MII/pH or SC monitoring was excluded.

Both MII/pH and SC monitoring were divided into 120-second intervals in order to assess the relationship between reflux events and stress. For each SC interval the peaks/second, AHP and ASP values were automatically calculated and stored in a database. The peaks/second value corresponding to the 75th percentile was calculated and the SC intervals were considered positive for the presence of stress if their peaks/second value was included in the fourth quartile range.

SC intervals that included the onset of a reflux event were defined as GER-associated, and these were considered positive for stress according to the peaks/second values described above. Duration, proximal extent and pH of stress-positive and stress-negative GER-associated SC intervals were compared. Peaks/second, AHP and ASP values of GER-associated and non-GER-associated SC intervals were also compared.

### Statistical Analysis

The distribution of all continuous variables was assessed by the Shapiro-Wilk test and by graphic analysis. The Mann-Whitney *U* test was used for comparison between groups. Statistical significance was set at *p*<0.05.

The symptom association probability (SAP) and the symptom sensitivity index (SSI) were calculated to establish the association between reflux events and stress for each infant [28]. Values of SAP>95% and SSI>10% were considered statistically significant. All results are shown as median and range, or median and interquartile range (IQR), unless otherwise indicated.

Statistical analysis was performed using the SPSS software package for Windows SPSS Inc., 2006, release 15.0, Chicago, Illinois, USA.

## Results

### Patients

Sixteen infants (seven males) were finally included in the study and underwent combined MII/pH and SC monitoring. Four infants were excluded as they had an insufficient duration of artifact-free monitoring (three infants with valid SC monitoring<6 hours; one infant whose signal from one of the six MII channels was interrupted after a few hours). The characteristics of the 12 remaining infants (six males) who completed the combined monitoring are reported in Table 1.

**Table 1:** General characteristics of the study population.

Case	Gestational age	Age	Post-conceptional age	Weight at delivery	Length at delivery	Weight at enrollment	Length at enrollment
number	(weeks)	(days)	(weeks)	(g)	(cm)	(g)	(cm)
1	39	18	41.6	3440	48.8	3500	49
2	40	32	44.6	3830	50.2	4370	54
3	34	44	40.3	2250	44	3300	49
4	38	28	42.0	3390	49	3720	53.5
5	37	21	40.0	3320	51	3250	51.5
6	33	45	39.4	1800	42.2	3310	48
7	40	17	42.4	2910	47	3250	49
8	38	37	43.3	2470	47.1	3540	52.5
9	39	21	42.0	3080	50	3500	54
10	38	29	42.1	3000	49.5	3910	53
11	41	32	45.6	3090	49.1	4040	54
12	39	34	43.9	3310	34	3530	54
**Median**	**38.5**	**30.5**	**42.0**	**3085.0**	**48.9**	**3515.0**	**52.7**
**Range**	**33.0–41.0**	**17.0–45.0**	**39.4–45.6**	**1800–3830**	**34.0–51.0**	**3250–4370**	**48.0–54.0**

### Combined MII/pH and SC Monitoring

The total artifact-free combined MII/pH and SC monitoring suitable for the present analysis amounted to 194.38 hours. The median (IQR) recording time per infant was 17.78 (11.53–20.38) hours. A total of 584 reflux events were detected by MII/pH, of which 100 (17.12%) were acid, 482 (82.53%) were weakly acidic and 2 (0.34%) were weakly alkaline.

SC monitoring was divided into 120-second intervals, for a total of 5,829 intervals. The median (IQR) peaks/second value was 0.03 (0.00–0.11) Hz; AHP was 1.42 (0.00–15.89) µSs, and ASP was 1.96 (0.00–18.02) µSs.

In order not to use any preset cut-off value for discomfort, the 75^th^ percentile peaks/second value, which was 0.11 peaks/second, was chosen. A total of 1418 (24.47%) SC intervals with a peaks/second value greater than the 75^th^ percentile were considered positive for the presence of stress. The median (IQR) peaks/second, AHP and ASP values for this dataset were 0.22 (0.16–0.32) peaks/second, 40.26 (13.06–143.49), and 47.24 (19.64–135.26) µSs, respectively. Combined MII/pH and SC monitoring data are illustrated per infant in Table 2.

**Table 2:** Combined esophageal multichannel intraluminal impedance/pH and skin conductance monitoring results. Data are presented as median (interquartile range, IQR).

Case	Reflux frequency	IBEI	Reflux duration	Reflux proximal extent	Peaks/second	AHP	ASP
number	(n/hr)	(%)	(s)	(cm)	(Hz)	(µSs)	(µSs)
1	3.96	2.05	9.1 (6.4–23.4)	5.0 (4.0–5.0)	0.01 (0.00–0.06)	0.00 (0.00–10.07)	0.00 (0.00–13.48)
2	0.82	0.31	10.8 (5.3–19.0)	4.0 (4.0–4.8)	0.03 (0.01–0.08)	1.90 (0.00–14.10)	2.62 (0.00–14.98)
3	1.33	1.09	13.0 (6.1–40.3)	4.0 (3.0–4.0)	0.16 (0.07–0.28)	17.18 (3.95–69.14)	23.61 (5.58–105.32)
4	7.94	6.88	16.6 (10.8–29.2)	5.0 (5.0–5.0)	0.01 (0.00–0.03)	0.00 (0.00–1.20)	0.00 (0.00–1.50)
5	3.65	3.63	18.2 (10.6–36.1)	4.5 (4.0–5.0)	0.00 (0.00–0.01)	0.00 (0.00–0.00)	0.00 (0.00–0.00)
6	3.63	1.55	8.9 (6.4–11.9)	5.0 (5.0–5.0)	0.02 (0.00–0.05)	0.52 (0.00–5.64)	0.63 (0.00–6.68)
7	2.65	1.25	8.3 (5.5–16.9)	5.0 (3.0–5.0)	0.03 (0.00–0.08)	1.10 (0.00–8.56)	1.75 (0.00–10.01)
8	3.74	2.29	11.6 (7.9–20.4)	5.0 (4.0–5.0)	0.05 (0.01–0.11)	2.23 (0.00–27.84)	3.63 (0.00–24.59)
9	1.66	1.24	12.3 (9.9–22.0)	4.0 (3.0–4.8)	0.03 (0.00–0.10)	0.69 (0.00–14.42)	0.96 (0.00–20.22)
10	2.26	1.12	14.9 (9.8–23.9)	4.0 (4.0–5.0)	0.03 (0.00–0.06)	0.62 (0.00–4.96)	0.73 (0.00–4.48)
11	2.92	2.96	20.0 (9.0–36.1)	4.0 (3.0–5.0)	0.07 (0.02–0.33)	5.7 (0.17–50.47)	9.45 (0.23–51.93)
12	5.74	3.38	14.7 (8.5–26.6)	5.0 (4.0–5.0)	0.07 (0.01–0.20)	3.21 (0.00–12.85)	4.98 (0.00–12.81)
**Median**	**3.27**	**1.80**	**12.8**	**5.0**	**0.03**	**1.42**	**1.96**
**IQR**	**2.11–3.80**	**1.21–3.07**	**7.8–26.7**	**4.0–5.0**	**0.00–0.11**	**0.00–15.89**	**0.00–18.02**

IBEI: impedance bolus exposure index, AHP: area huge peak; ASP: area small peak.

### Correlation Data

Five hundred fifteen reflux events (88.18%) were temporally associated with SC intervals with peaks/second-positive values (i.e., SC values>0). Only 69 (11.82%) reflux events were not associated with any SC signal variation. Two hundred nine (35.78%) of the observed GER-associated SC intervals were positive for stress, 34 of which (16.27%) were acid and 175 (83.73%) were weakly acidic, and none of which were weakly alkaline. The acid reflux rate, duration and proximal extent were similar in both the stress-positive and stress-negative group of reflux events (*p*>0.05). The association between reflux events and stress established with SAP and SII was statistically significant in 12/12 and 11/12 infants, respectively. The median (IQR) SAP% and SSI% were 97.75 (96.02–99.94) and 32.82 (22.32–66.74), respectively.

**Figure 2:**
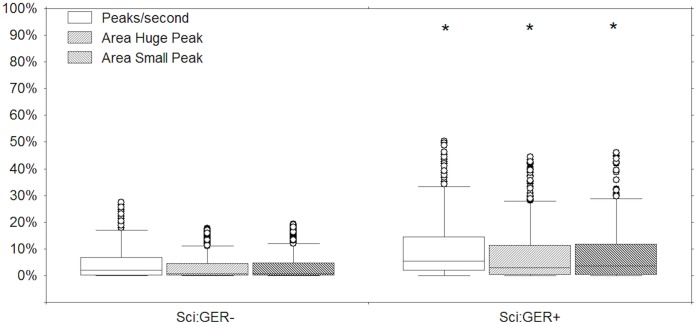
Peaks/second, area huge peak and area small peak normalized values in non-GER-associated skin conductance intervals (SCi:GER-) vs GER-associated skin conductance intervals (SCi:GER+). * Mann-Whitney test: *p*<0.001.

**Figure 3:**
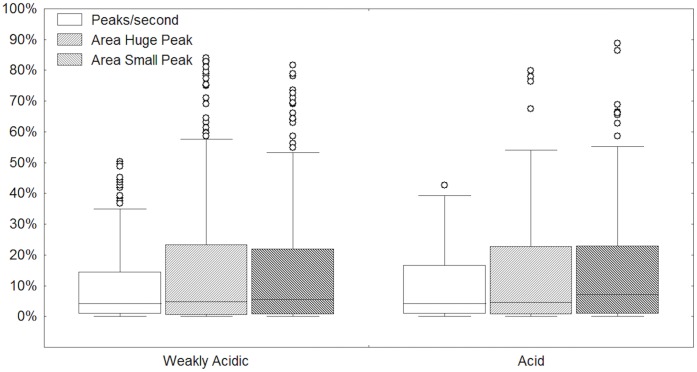
Peaks/second, area huge peak and area small peak normalized values in skin conductance monitoring intervals associated to acid and weakly acidic reflux events.

Finally, peaks/second, AHP and ASP in non-GER-associated and GER-associated SC intervals were compared. All three SC interval variables were increased in weakly acidic GER-associated SC intervals compared to non-GER-associated SC intervals (Figure 2), whereas no difference was found for the same variables between acid and weakly acidic GER-associated SC intervals (Figure 3).

## Discussion

This is the first study to investigate the relationship between reflux events and discomfort in infants suffering from GERD using combined MII/pH and SC monitoring. Strength of this study includes the novelty of combining these two techniques for 20 consecutive hours in an attempt to objectively measure the discomfort produced by common occurrences such as reflux events in the early stages of life.

Our primary finding is the significant association between reflux events and increased SC peaks/second values, which was observed in all the infants evaluated. Further, both weakly acidic and acid reflux events were shown to be equally responsible for discomfort, suggesting that pH might not be the main cause of stress in GERD infants. The suitability of antacid drug prescription in the management of GERD in the first months of life is currently openly debated. Our results raise more new questions as to the appropriateness of antacid management protocols in infants with GERD [29].

According to van der Pol et al up to two-thirds of reflux events detected by MII/pH monitoring in infancy are weakly acidic [30]; their clinical relevance is probably significant, though poorly considered until now due to the limited possibility of investigation. International guidelines suggest that only infants with acidic reflux events should be treated [3], [4], while weakly acidic reflux events should not be considered pathological. On the other hand, the introduction of MII/pH has confirmed that weakly acidic reflux events are common and occur particularly after meals, suggesting an association with some clinical features [8], [31], [32]. Thus the role of MII in improving diagnoses, which in turn leads to more appropriate treatment, should be underlined [33], [34]. Many infants suffering from GERD are empirically treated with acid-suppression medications such as ranitidine and proton-pump inhibitors. Indeed, the use of such pharmacologic agents in children has recently increased enormously worldwide [9], [35]–[37], despite recent reports showing that proton-pump inhibitors are not effective in reducing GERD symptoms in infants [30]. As a consequence, the majority of infants who are prescribed antireflux drugs do not meet the diagnostic criteria for GERD and probably don’t need empirical antacid therapy. This highlights the importance of assessing of the clinical role of weakly acidic reflux events as a mandatory first step in the improvement of GERD therapy in infants, who can’t express clearly their discomfort.

In order to do this, our study combined two advanced techniques: MII/pH and SC monitoring. MII/pH monitoring is more accurate than pH monitoring alone in the evaluation of reflux events, as it determines duration and proximal extent of each event independently of pH [32]. The latter provides a measure of discomfort by analyzing changes in SC [38], [39]. This technique has been developed in the last decade and has been favorably used to assess discomfort in newborns, infants and children undergoing painful or invasive procedures, such as artificial ventilation and heel stick [17], [20]–[22], [40]. Until now SC has only been recorded for short periods of time (minutes or a few hours). For the first time, we have performed SC monitoring, in combination with another monitoring technique, for 20 consecutive hours in each infant. Given the length of monitoring, we had to elaborate a software that would synchronize and properly evaluate the data obtained from both monitoring techniques. The monitoring sessions were divided into 120-second intervals, which were checked for the presence of reflux and stress; a 2-minute duration was considered appropriate to evaluate the relationship between different combined recorded events [15].

The main variable of SC interval used to assess the relationship between reflux events and discomfort was peaks/second, which has been reported to be the best indicator of discomfort and pain in infants [17, 14 20, 21]. On the other hand, Pereira da Silva et al found that ASP and AHP reacted more than peaks/second during prolonged painful procedures [15].

In our study, the SC intervals with a peaks/second value in 75th percentile or greater were considered positive for stress. This cut-off was chosen arbitrarily, as no previous data from similar studies are available, and it allowed us to identify the intervals associated with higher stress and with higher clinical relevance. Interestingly, this value was 0.11 peaks/sec, similar to the cut off value used by Ledowski to define moderate and severe pain postoperatively in adults and children [39], [41]. The SC index may be influenced by movement artefacts and the electrodes should be fastened to the extremity by wrapping to reduce such artifacts [42]. Prolonged monitoring sessions possibly increase the artefact rate in particular in those newborns showing increased crying time and fussiness independently from GERD. This might be a limit for routinely application of SC monitoring in clinical practice, which can be improved by the avoidance of unnecessary manipulation, constant environmental conditions and further training of the operator. Moreover, the evaluation of clinical symptoms during the combined monitoring is intended to be performed in further studies to assess the clinical relevance of stress-associated reflux events.

The main result is the significant association between reflux and stress observed both in the entirety of the study population, as well as in the SAP and SSI values for each infant. Secondly, there was no difference between weakly acidic and acid reflux events in terms of SC values. This result is relevant as it shows that weakly acidic reflux events (which represent the majority of reflux events in infancy, as reported also in our study population) are stressful for infants, and the discomfort they cause is similar to that induced by acid reflux events. Anyway, SC monitoring with long-time recording is still an experimental method and it is not yet validated for clinical practice because of the limits reported above. If our results are confirmed in a larger population along with symptoms recording, the diagnostic and therapeutic methods currently applied to infants with GERD should be revisited, as weakly acidic events would also deserve appropriate therapy [9]. This would suggest that pH monitoring should always be used in combination with MII in order to detect the frequency, characteristics and quantity of non-acid reflux events [31]. New therapeutic devices that may reduce the reflux events themselves, instead simply of reducing their acidity, should also be assessed.

### Conclusions

This is the first study performed to detect discomfort in infants suffering from GERD using combined MI/pH and SC monitoring for 20 consecutive hours. Both weakly acidic and acid reflux events have been shown to be equally responsible for discomfort, as measured by variations in SC values. These results may raise some concerns about the over-prescription of antacid drugs in infancy.
